# SERUM LEPTIN, ATHEROGENIC LIPIDS AND GLUCOSE LEVELS IN PATIENTS WITH SKIN TAGS

**DOI:** 10.4103/0019-5154.48980

**Published:** 2009

**Authors:** Canan Gorpelioglu, Emel Erdal, Yasemin Ardicoglu, Bahattin Adam, Evren Sarifakioglu

**Affiliations:** *Form the Department of Dermatology, Fatih University Faculty of Medicine, Ankara, Turkey*; 1 *Form the Department of Dermatology, Mesa Hospital, Ankara, Turkey*; 2 *Form the Department of Biochemistry, Mesa Hospital, Ankara, Turkey*; 3 *Form the Department of Biochemistry, Konya Hospital of The Turkish Health and Therapy Foundation, Konya, Turkey*

**Keywords:** *Atherogenic lipid*, *leptin*, *skin tags*

## Abstract

**Aim:**

To investigate the relationship between serum leptin, atherogenic lipid and glucose levels in patients with skin tags and healthy controls.

**Materials and Methods:**

A total of 58 patients, with at least three skin tags, aged 24 to 85 years, and 31 healthy controls aged 30 to 70 years, were examined in the present study. The subjects in all the groups were selected with statistically similar Body Mass Index (BMI). Fasting concentrations of plasma glucose, serum lipids including triglyceride, total cholesterol, and high-density lipoprotein cholesterol (HDL) and low-density lipoprotein cholesterol (LDL), HbA1c, and leptin were measured by enzyme-linked immunosorbent assay (ELISA). In addition, serum LDL level was calculated using Friedewald's formula.

**Results:**

There was no significant difference in age, sex, BMI, HbA1c, triglyceride, HDL and leptin levels between the groups. Skin tags group showed significantly higher levels of total cholesterol and LDL, when compared with the healthy controls groups (*P* < 0.01). In addition, regression analysis showed that leptin level was positively correlated to serum triglyceride level (r = 0.265, *P* = 0.044).

**Conclusion:**

Total cholesterol and LDL serum levels should be controlled in patients with skin tags. On the other hand, glucose, leptin and HbA1c serum levels may not be as important as is being considered in recent times.

## Introduction

Skin tags (ST) are small, soft, pedunculated, often pigmented lesions, usually occurring on the eyelids, neck and axillae. The condition is very common, particularly in middle-aged and elderly women. There have been a few reports in the literature that the presence of ST is associated with diabetes mellitus, obesity and atherogenic lipid profile.[1–5]

Leptin, a 16-kDa protein, which is a product of the obese gene, is involved in the regulation of appetite and energy expenditure. Leptin is produced mainly by adipocytes and low levels have been detected in gastric fundic epithelium, intestine and skeletal muscle. The protein circulates at concentrations proportional to body fat and decreases body weight by inhibiting food intake and inducing thermogenesis.[6] Recently, leptin has been reported to stimulate the proliferation of various cell types and is considered to be a new growth factor.[7,8] Leptin exerts a number of regulatory functions; most of them are poorly understood.[9] Plasma leptin displays a strong association with cardiovascular risk factors, including obesity, insulin resistance, hypertension, dyslipidemia, hyperuricemia, inflammatory markers.[10,11]

The present study was designed to investigate the relationship between serum leptin, atherogenic lipid and glucose levels in patients with skin tags and healthy controls.

## Materials and Methods

A total of 58 patients, with at least three skin tags, and 31 healthy controls were included in the study. Subjects with similar BMI were selected in all the groups. Skin tags were defined as fleshy, pedunculated soft lesions, skin colored or darker hue that were at least 0.2cm height and diameter. The height and weight of the patients were measured and BMI estimated. Body mass index was calculated by dividing body weight to height square (kg/m^2^). Patients were considered according to their BMI – BMI ≤ 18 as thin, BMI between 19 and 25 as normal, BMI between 26 and 29 as overweight and BMI ≥30 as obese. All the patients and the control group were informed about the aim and procedure of the study. All the blood samples of the patient and control group were taken after an eight-hour starvation, at 8:00-9:00 am.

The study protocol conformed to the ethical guidelines of the Declaration of Helsinki and was approved by the local Ethical Committee.

Exclusion criteria for the study were - patients receiving drugs with a known antihyperlipidemic effect, pregnant women and patients with thyroid function disorder.

Venous blood was taken from the patients and the control group. Sera were separated after centrifugation at 5000rpm for 5 minutes, stored at -20°C and thawed just before analysis. Leptin levels were measured by solid phase sandwich ELISA, using a commercial kit (Human Leptin, Biosource International, CA, USA). Sample for quality control was included in each assay. The intraassay and interassay coefficient of variations for the assay were 3.0% and 3.9% for the concentrations 147.5pg/ml and 150.6pg/ml, respectively. In addition, serum glucose, total cholesterol, HDL and HbA1c levels were measured by using Hitachi 902 Autoanalyser (Roche Diagnostics, Germany). Serum LDL levels were calculated using Friedewald's formula.

All the results were expressed as means ± (SD) values. The significance of the difference between the groups was assessed by unpaired Student's t-test or Mann Whitney U tests, for continuous variables. The chi-square test or Fischer's Exact test was used for testing prevalence between groups. The statistical analysis was performed using SPSS-11 programme and *P* values < 0.05 were considered significant.

## Results

A total of 58 patients with skin tags, aged 24 to 85 years (10 male, 48 female; median age 55.6) and 31 healthy controls aged 30 to 70 years (5 male, 26 female; median age 52.5) were examined in the present study. No patient had signs of acute infectious disease at the time of the study.

There was no significant difference in age, sex, BMI, HbA1c, triglyceride, high-density lipoprotein and leptin levels between the groups [Table 1].

**Table 1:** Lipid profile, leptin and glucose levels in the serum

	Patients (58)	Controls (31)	*P*
Total cholesterol (mg/dl)	215.8 ± 39.3	191.2 ± 33.1	0.004*
HDL (mg/dl)	56.2 ± 15.4	62.7 ± 16.8	0.071
LDL (mg/dl)	130.2 ± 36.6	105.5 ± 29.8	0.002*
VLDL (mg/dl)	27.9 ± 14.3	27.1 ± 21.3	0.840
Triglycerides (mg/dl)	139.4 ± 70.9	111.9 ± 52.1	0.064
Glucose (mg/dl)	102.4 ± 29.6	105.6 ± 28.7	0.625
HbA1c (%)	5.6 ± 0.8	5.4 ± 0.8	0.439
Leptin (pg/ml)	496.2 ± 266.2	392.5 ± 238.5	0.73

**Figure 1:**
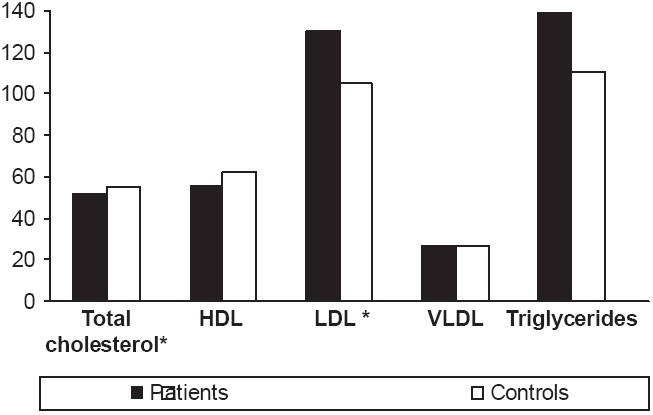
Lipid profiles of the groups

Skin tags group showed significantly higher levels of total cholesterol and LDL, when compared with the healthy controls group (*P* < 0.01) [Figure 1]. In addition, regression analysis showed that leptin levels were positively correlated with serum triglyceride level (r = 0.265, *P* = 0.044) [Table 1].

A correlation of serum leptin level with body mass and body mass index was found in both control and skin tag patient groups.

## Discussion

Skin tags are common benign lesions that are composed of loose fibrous tissue and which occur mainly on the neck and major flexures as small soft, pedunculated protrusions. These lesions are very common, particularly in women at menopause or later.[12] Obesity is a factor that has been associated with the development of ST.[13]

Multiple skin tags are frequently associated with non-insulin-dependent diabetes mellitus (NIDDM) and obesity.[1,2] In our study, the mean BMI of our patients was 27.7 ± 4.9; they were mostly overweight. There was no significant difference in HbA1c, and serum glucose levels between the groups. Kahana[1] and Agarval[2] found a relationship between DM and ST. In another study, Rasi *et al.*[3] investigated oral glucose tolerance test (OGTT) with 75g glucose and showed an increased risk of diabetes mellitus in patients with ST. However, we could not find this kind of relationship in our study, and this might be because we did not test our patients for OGTT. Our aim was especially to study the relationship between lipid metabolism, serum leptin level and ST. Our control group had a similar BMI (28.7 ± 7.9); therefore, we thought that the relationship between ST and DM might be associated with the patients having obesity and obesity related glucose intolerance. Similarly, in another study, Marthur *et al*. estimated insulin resistance in 10 patients with multiple ST and 10 control subjects matched for age, sex and body weight. They concluded that ST are not markers of insulin resistance and it is possible that epidermal growth factor or other growth factors may play a role in the pathogenesis of the ST.[4]

There are only a few numbers of studies about the relationship between ST and atherogenic lipid profile. Crook investigated serum lipid profile in four patients with ST and found increased serum triglyceride and decreased HDL cholesterol.[5] In yet another study, Erdogan *et al.* found increased total cholesterol in 36 patients with ST, when compared with 22 healthy controls.[6] In our study, total cholesterol levels and LDL serum levels were higher in patients with ST, when compared with the healthy control group.

Leptin, a major adipocyte-derived hormone, is involved in the regulation of food intake and energy expenditure.[7] Leptin reduces food intake by up-regulating anorexigenic neuropeptides. Other leptin actions include those related to glucose metabolism, bone physiology, angiogenesis, and immunocompetence.[14,15]

How leptin plays a role in development of the ST is not known. However, there are some studies that might be helpful in the etiopathogenesis. Frank *et al*. investigated the beneficial effect of leptin on the proliferation of cutaneous keratinocytes in rodents.[16] Moreover, direct proliferative effects of leptin on mouse and human keratinocytes have also been reported.[17] The impact of leptin as a mitogenic factor in skin repair has been intensively studied.[18] However, the molecular mechanism underlying the cell growth-stimulatory effect of leptin is not totally understood.

Leptin circulates at concentrations proportional to body fat and decreases body weight by inhibiting food intake and inducing thermogenesis.[7] Our hypothesis was that, if a leptin deficiency can be found, ST formation can be prevented in patients, maybe in the future, with leptin treatment. Consequently, in this study, we investigated the relationship between serum leptin levels in patients with ST and healthy controls. In order to eliminate the effect of adipose tissue (as leptin is being secreted from adipose tissue), we chose our control group to include patients with similar BMI. No statistically significant difference was found in the serum leptin levels in both groups.

As a result, patients with skin tags were found to have significantly high total cholesterol and LDL serum levels, when compared with the healthy control group. However, there was no statically significant difference in the serum glucose, leptin, and HbA1c levels in both the groups.

To conclude, total cholesterol and LDL serum levels should be controlled in patients with skin tags. On the other hand, glucose, leptin and HbA1c serum levels may not be as important it is being considered in recent times.
